# Effects of a personalized PERMA-based intervention on the mental health of junior high school students: a randomized clinical trial

**DOI:** 10.3389/fpsyg.2025.1535744

**Published:** 2025-08-29

**Authors:** Lei Zhang, Xiao Chen, Hengjing Wan

**Affiliations:** ^1^Shanghai Minhang District Mental Health Center (Pujiang Hospital of Shanghai Mental Health Center), Shanghai, China; ^2^School of Nursing, Fudan University, Shanghai, China

**Keywords:** positive psychology interventions, PERMA, adolescent health, well-being, resilience, coping styles

## Abstract

**Background:**

Adolescence is a critical period marked by increasing academic pressure and evolving self-cognition, making junior high school students particularly vulnerable to mental health challenges. Positive psychology interventions (PPIs) based on the PERMA framework have shown promise in improving well-being, but studies targeting Chinese middle school students remain limited. This study aimed to evaluate the effectiveness of a personalized PERMA-based PPI in enhancing well-being and resilience among junior high school students in Shanghai, China.

**Methods:**

A total of 154 students were randomly assigned to either an experimental group (*n* = 77) or a control group (*n* = 77). The experimental group participated in an eight-week intervention involving weekly sessions designed to enhance the five dimensions of the PERMA framework: Positive Emotion, Engagement, Relationships, Meaning, and Accomplishment. The control group continued with regular psychology courses. Pre- and post-intervention assessments were conducted using validated scales measuring well-being, resilience, and coping styles. Paired *t*-tests were used for within-group comparisons and ANCOVA for between-group comparisons, analyzed in R (v4.2.2).

**Results:**

At baseline, the experimental group had lower scores in positive emotion (12.4 ± 4.02 vs. 13.8 ± 4.39), relationships (12.4 ± 4.22 vs. 13.8 ± 4.05), and total well-being (47.5 ± 13.6 vs. 51.1 ± 14.4) compared to the control group. After the intervention, the experimental group showed significant improvements in positive emotion (+2.3), relationship (+2.2), and total well-being (+3.4). The experimental group also demonstrated significant increases in accomplishment (+1.4), engagement (+1.2), goal focus (+2.1), positive cognition (+1.5), and total resilience (+5.2). No significant changes were observed in coping styles, though slight improvements in positive coping tendency (from −0.02 to −0.13) and a slight decrease in negative coping tendency (from −0.02 to −0.01).

**Conclusion:**

This study provides empirical evidence for the effectiveness of personalized PPIs in addressing adolescents’ mental health challenges, with notable improvements in well-being and resilience. Limitations include the small sample size for paired analysis (*n* = 58) and the timing of the intervention during the final exam period, which might have introduced biases. Future research should explore the sustained effects of such interventions and their scalability across diverse educational settings.

## 1 Introduction

Adolescence, identified by the World Health Organization (WHO) as a critical period for psychological development, is often marked by mental health challenges (World Health Organization, 2021). Global estimates indicate that 6.2% of adolescents experience depression, and 6.5% suffer from anxiety (Erskine et al., 2017). These mental health challenges profoundly impact adolescents’ well-being and academic performance, disrupting psychosocial functioning, diminishing quality of life, and contributing to poor school attendance (Finning et al., 2019).

Middle school students, in particular, are vulnerable to these mental health issues. This period is characterized by rapid physical and psychological changes, coupled with the challenges of academic demands, the complexities of peer relationships, and the quest for self-identity (Wu et al., 2022). In the context of China, the aftermath of the COVID-19 pandemic has further exacerbated these challenges, with reported prevalence of insomnia, depressive symptoms, and anxiety among Chinese adolescents at 37.80, 48.20, and 36.70%, respectively (Chi et al., 2021). These issues not only disrupt students’ academic performance and daily lives but may also lead to long-term psychological issues, potentially affecting their social adjustment in adulthood (Hankin et al., 2007). Consequently, there is an urgent need for effective mental health interventions to enhance the psychological well-being of middle school students in China.

The PERMA model, developed by Seligman (2011), has gained recognition in the field of positive psychology. This model encompasses both hedonic components (such as happiness, life satisfaction, the presence of positive affect, and the absence of negative affect) and eudaimonic components (including positive psychological functioning based on indicators like meaning in life and positive relationships), and defines well-being through five dimensions: Positive Emotion, Engagement, Relationships, Meaning, and Accomplishment. In Western educational settings, studies have demonstrated that interventions based on the PERMA framework can improve adolescents’ overall well-being by fostering positive emotions, enhancing engagement in activities, strengthening relationships, and increasing a sense of purpose (Eisenreich, 2023; Jie et al., 2022). However, research applying the PERMA framework in Chinese educational contexts is sparse. Given that Chinese culture places a strong emphasis on academic achievement and social harmony, the dimensions of the PERMA framework—particularly the focus on engagement, relationships, and accomplishment—are highly relevant. However, these cultural factors, including collectivist values and the prioritization of academic success, may influence how such interventions are received and their potential effectiveness.

Previous studies have primarily focused on the effectiveness of the PERMA model in Western contexts, but less attention has been paid to how these dimensions resonate within Chinese middle school students. Additionally, while studies have explored the general benefits of positive psychology interventions (Lyubomirsky et al., 2011; Norrish et al., 2013), the application of these interventions in China’s academic-centric environment remains underexplored. This study aims to fill these gaps by evaluating the impact of a PERMA-based Positive Health Intervention on the psychological well-being of Chinese middle school students.

In particular, this research will focus on three key indicators: the Multidimensional Happiness Scale, the Psychological Resilience Scale, and Coping Tendency. By fostering positive emotions, enhancing engagement in school activities, improving social relationships, cultivating a sense of meaning, and strengthening academic achievement, the intervention aims to promote students’ overall psychological well-being. This study not only contributes empirical evidence supporting the use of school-based mental health interventions in China but also provides insights into how such interventions can be adapted to fit the unique cultural and educational characteristics of the Chinese context. By addressing the specific challenges Chinese adolescents face, this research offers valuable implications for the development of policies and practices aimed at improving adolescent mental health.

## 2 Methods and materials

### 2.1 Participants

From March to June 2024, this study was conducted in a junior high school in Minhang District, Shanghai, China. Participants were selected using a random number generator to choose four seventh-grade classes for the study. Two classes were randomly assigned to the experimental group, while the remaining two served as the control group. The intervention was implemented on a class-by-class basis. Students were excluded if they were unable to attend regularly due to reasons such as sick leave, vacations, or personal leave; if they were already participating in other psychological intervention studies; if they chose to withdraw during the study; or if they had a diagnosed mental illness. Both the parents and the students were fully informed about the study, provided consent to participate, and signed informed consent forms. To ensure participant anonymity, each student was assigned a unique random number, known only to them, for the completion of all subsequent evaluations. This study was approved by the Ethics Committee of Shanghai Minhang Mental Health Center (LW202304).

*A priori* power analysis was conducted using G*Power 3.1 with an effect size of 0.5, an alpha level of 0.05, and a power of 0.8, suggesting a required total sample size of 128 participants (Kang, 2021). Accounting for a potential dropout rate of 15%, the study aimed to recruit 152 participants, with 76 in each group, to ensure adequate sample size.

### 2.2 Pre-intervention preparation

The intervention course working team consisted of 10 members with diverse expertise: 2 psychology experts who provided guidance on the intervention program’s content, 1 psychiatric nurse expert who offered support for handling special situations during the intervention activities, 4 psychological counselors/psychotherapists who facilitated the intervention activities for the experimental group, 2 psychiatric nurses (not involved in intervention activities) who distributed and collected questionnaires after undergoing standardized training, and 1 graduate nursing student and 1 of the psychiatric nurses who were responsible for organizing, verifying, and entering the questionnaire data.

Before the intervention course commenced, the psychological status of participants in the experimental group was assessed to identify their specific needs for psychological course guidance. The assessment explored their primary objectives for participation, emotional distress, interpersonal relationship challenges, academic difficulties, feelings of meaninglessness, and lack of achievement. Based on the survey results, the course content was tailored and adjusted on a class-by-class basis to better address the identified needs during the subsequent intervention sessions. For example, students who reported high academic stress were provided with engagement-focused sessions aimed at enhancing their involvement in school activities, using strategies such as goal-setting exercises and mindfulness techniques to alleviate stress and improve concentration. In addition, students experiencing interpersonal difficulties received guidance on relationship-building through structured group activities designed to foster communication, empathy, and cooperation. These adjustments were implemented to ensure that each student received relevant support tailored to their individual needs, helping to maximize the effectiveness of the intervention.

### 2.3 Intervention program

The intervention program was based on the five dimensions of PERMA (positive emotion, engagement, relationships, meaning, and accomplishment), and assessments were designed to focus on enhancing these core aspects of well-being. The course consisted of one session per week, each lasting 45 min, for a total of 8 weeks. The control group attended only the standard on-campus psychology curriculum and did not participate in any positive psychology group intervention activities based on the PERMA theory. In contrast, the experimental group received a tailored positive psychology group intervention program structured around the PERMA framework, which included sessions on Introduction, Positive Emotions, Positive Engagement, Positive Relationships, Positive Achievements, Positive Meaning, and a concluding session titled Farewell. The standard program schedule was listed in Supplementary Table S1.

Each session followed a consistent structure, comprising warm-up activities, theme-based exercises, review and summary, and assigned homework. Various teaching strategies were utilized, including group discussions, interactive games, multimedia presentations, question-and-answer segments, scenario interpretations, and case analyses.

The intervention program addressed several key themes. Participants were guided to understand their emotional characteristics and recognize the influence of emotions on behavior and life. They learned to observe others’ feelings, express their emotions appropriately, and employ techniques to maintain positive emotions while effectively releasing negative emotions. Mindfulness exercises were incorporated to enhance focus and awareness of the present moment. The program emphasized identifying sources of happiness within the learning process and developing effective communication skills to improve interactions with parents. Participants assessed their ability to form and maintain friendships and explored strategies to enhance peer relationships. Reflective exercises on significant life events helped participants identify patterns of optimism or pessimism and challenge irrational beliefs to improve self-evaluation. Additionally, the program guided participants in recognizing their personal strengths and setting realistic, stage-appropriate life goals.

This structured and multi-faceted intervention was designed to promote well-being across multiple dimensions, in alignment with the PERMA framework.

### 2.4 Questionnaire assessment

At baseline, a general information questionnaire was administered to collect data on class, gender, age, parental relationships, peer relationships, personality traits, life satisfaction, dietary habits, and physical activity levels. Before and after the intervention, three questionnaires were utilized to assess the psychological status of the participants and evaluate the effectiveness of the course intervention.

The Plural Well-being Questionnaire for Middle School Students was developed by a Chinese scholar to assess “feeling good” and “performing well” among middle school students based on the PERMA theory. This questionnaire comprises four dimensions: positive emotions, engagement, accomplishment, and relationships. Each dimension includes four questions, resulting in a total of 16 items. Responses are scored using a 5-point Likert scale, where 1 represents “very inconsistent with me” and 5 represents “very consistent with me.” Scores range from 1 to 5 points for each question, with a total possible score of 16–80 points. Higher scores indicate higher levels of multidimensional well-being. The questionnaire was validated in a sample of 663 junior and senior high school students from Hebei and Shandong provinces. Its internal consistency reliability, measured by Cronbach’s α coefficient, was 0.909, indicating high reliability (Shang, 2014).

The Adolescent Resilience Scale, developed by Hu Yueqin and Gan Yiqun in 2008, assesses resilience across two dimensions: personal strength and support. The personal strength dimension includes three factors: goal focus, emotional control, and positive cognition; while the support dimension encompasses two factors: family support and interpersonal assistance. The scale comprises 27 items, with a total Cronbach’s α coefficient of 0.83, indicating good reliability (Hu and Gan, 2008; Jiao et al., 2022).

The Simple Coping Style Scale, created by Xie Yaning in 1998, is an adaptation of the Ways of Coping Questionnaire developed by Folkman (Zeidner and Endler, 1996). It was designed to reflect the relationship between coping styles and mental health. The scale includes two subscales: positive coping tendency and negative coping tendency, with a total of 20 items. Each item is rated on a four-point scale: “not used” (0 points), “occasionally used” (1 point), “sometimes used” (2 points), and “often used” (3 points). Higher scores in positive coping indicate lower levels of psychological problems and symptoms, while higher scores in negative coping suggest greater psychological difficulties. The scale has a total Cronbach’s α coefficient of 0.90, demonstrating excellent reliability. The coping tendency is calculated using the formula: Coping tendency = Z (positive coping) - Z (negative coping), where Z is the standard score calculated using the mean and standard deviation of the positive and negative coping scores. If the coping tendency score is greater than 0, it indicates a preference for positive coping strategies under stress. Conversely, a score less than 0 suggests a tendency toward negative coping strategies (Xie, 1998).

### 2.5 Statistical analysis

After data entry by two individuals, statistical analyses were performed using R software (v4.2.2). Normally distributed continuous data were expressed as mean ± standard deviation, while non-normally distributed continuous data were presented as medians with interquartile ranges. Categorical data were described using frequencies and percentages. For between-group comparisons, chi-square tests were used for unordered categorical data. Normally distributed continuous data were analyzed using two independent sample t-tests or analysis of covariance (ANCOVA) with adjustments for covariates. Non-normally distributed continuous data were analyzed using the Wilcoxon rank-sum test. For paired data, a linear mixed-effects model was used, with intervention and potential covariates set as fixed effects and individual IDs treated as random effects.

In addition to analyzing changes in continuous scores of the well-being and resilience scales, we further categorized the scores to examine the proportion of students who reached different threshold levels. Chi-square tests were conducted to compare the proportions of students in the intervention and control groups who scored above specific cutoff points (e.g., ≥10, ≥11, ≥12, etc.) on each variable. This categorical comparison aimed to assess whether the PERMA-based intervention increased the proportion of students reaching higher levels of well-being or psychological resilience, suggesting potential meaningful thresholds for future intervention study (Kunkle et al., 2020; Lipsey et al., 2012). A *p*-value < 0.05 was considered statistically significant.

## 3 Results

### 3.1 Baseline characteristics of study population

A total of 154 students participated in the study, with two classes (*n* = 77) assigned to the experimental group and two classes (*n* = 77) to the control group. All students completed the full intervention course and both the pre- and post-intervention assessments. As shown in Table 1, the mean age was 13 years, and gender distribution was similar between groups (48.1% male in the experimental group vs. 55.8% in the control group). Most participants lived with their parents in typical three-person households and reported being satisfied with their parental relationships. Over 90% of the participants reported having at least one close friend, maintaining a normal or healthy diet, and engaging in physical activity at least once per week.

**Table 1:** Baseline characteristics of study population.

Characteristics	Experimental group (*N* = 77)	Control group (*N* = 77)	*p*-value^a^
Sex			0.333
Male	37 (48.1%)	43 (55.8%)	
Female	40 (51.9%)	34 (44.2%)	
Age (year)	13.0 ± 0.58	13.0 ± 0.79	0.728
Satisfaction with the relationship between parents			0.372
Very satisfied	19 (24.7%)	30 (39.0%)	
Satisfied	28 (36.4%)	25 (32.5%)	
Generally satisfied	20 (26.0%)	13 (16.9%)	
Not satisfied	6 (7.8%)	6 (7.8%)	
Very not satisfied	4 (5.2%)	3 (3.9%)	
Long-term roommates			0.672
Parents	56 (72.7%)	62 (80.5%)	
Mother	13 (16.9%)	8 (10.4%)	
Father	4 (5.2%)	4 (5.2%)	
Grandparents	4 (5.2%)	3 (3.9%)	
Family structure			0.828
Three generations	24 (31.2%)	29 (37.7%)	
Core family^b^	39 (50.6%)	34 (44.2%)	
Single-parent family	9 (11.7%)	10 (13.0%)	
Divorced and reorganized family	5 (6.5%)	4 (5.2%)	
How many good friends do you think you have?			0.081
None	9 (11.7%)	3 (3.9%)	
1 ~ 2	26 (33.8%)	20 (26.0%)	
≥3	42 (54.5%)	54 (70.1%)	
Recent dietary status			0.622
No intention to eat	6 (7.8%)	4 (5.2%)	
Normal	38 (49.4%)	35 (45.5%)	
Great	33 (42.9%)	38 (49.4%)	
Frequency of physical activity			0.603
0 times per week	1 (1.3%)	4 (5.2%)	
1 ~ 2 times per week	21 (27.3%)	19 (24.7%)	
3 ~ 4 times per week	23 (29.9%)	25 (32.5%)	
≥5 times per week	32 (41.6%)	29 (37.7%)	

Data are presented as number (percentile) or mean ± SD.

a Group difference was tested by *t* test for continuous variables and chi-square test or Fisher test for categorical variables.

b Core family means children live with their parents.

The pre-intervention survey revealed that the primary motivations for participating in psychological counseling were to learn psychological skills (64.3%) and address personal psychological issues (62.9%). The most common challenges reported were high academic pressure or lack of motivation to study, followed by difficulties in interpersonal relationships. To address these concerns, the intervention program included two sessions focused on active engagement, designed to help students improve their concentration and discover ways to derive happiness from the learning process. Additionally, two sessions were dedicated to positive relationships, aimed at improving participants’ interactions with parents and peers.

### 3.2 Well-being outcomes

**Figure 1:**
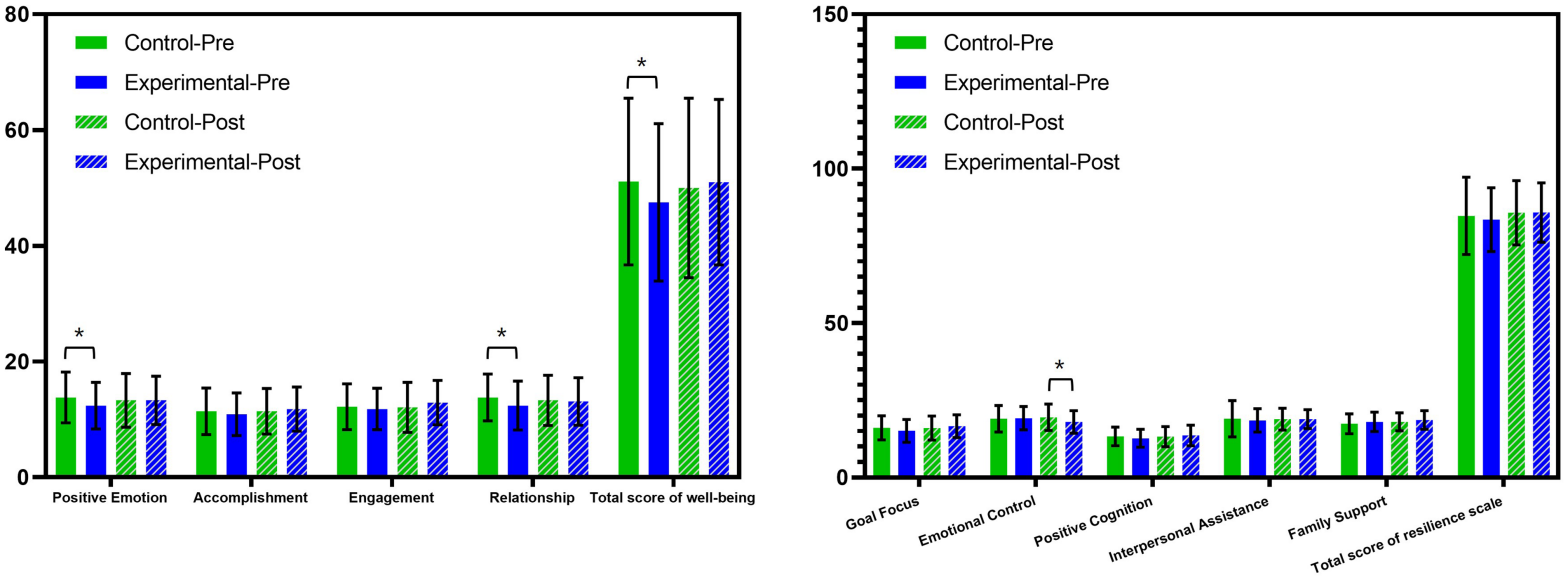
Scores of well-being (left) and resilience (right) of the control group and experimental group pre and post intervention in the whole population (*N* = 154. Data are presented as mean and SD. *: significant difference between groups tested by ANCOVA).

At baseline, the control group had significantly higher scores than the experimental group for positive emotion (13.8 ± 4.39 vs. 12.4 ± 4.02), relationship (13.8 ± 4.05 vs. 12.4 ± 4.22), and total well-being (51.1 ± 14.4 vs. 47.5 ± 13.6) as shown in Figure 1 and Table 2. After the intervention, no statistically significant differences were observed between the two groups across all five variables.

**Table 2:** Comparison of scores between experimental group and control group before and after the intervention.

Variable	Timepoint	Experimental group	Control group	*P*-value1	*P*-value2
**Total score of well-being**	Pre	47.5 ± 13.6	51.1 ± 14.4	0.110	**0.047**
	Post	51.0 ± 14.3	50.0 ± 15.5	0.683	0.607
Positive emotion	Pre	12.4 ± 4.02	13.8 ± 4.39	**0.040**	**0.016**
	Post	13.3 ± 4.19	13.3 ± 4.64	0.956	0.947
Accomplishment	Pre	10.9 ± 3.67	11.4 ± 4.02	0.381	0.296
	Post	11.8 ± 3.80	11.4 ± 3.94	0.510	0.451
Engagement	Pre	11.8 ± 3.58	12.2 ± 3.95	0.609	0.561
	Post	12.9 ± 3.83	12.1 ± 4.32	0.216	0.134
Relationship	Pre	12.4 ± 4.22	13.8 ± 4.05	**0.041**	**0.015**
	Post	13.1 ± 4.12	13.3 ± 4.34	0.747	0.696
**Total score of resilience**	Pre	83.5 ± 10.3	84.7 ± 12.5	0.514	0.508
	Post	85.8 ± 9.62	85.7 ± 10.4	0.949	0.949
Goal focus	Pre	15.1 ± 3.67	16.1 ± 3.89	0.127	0.105
	Post	16.6 ± 3.69	16.0 ± 3.93	0.302	0.250
Emotional control	Pre	19.2 ± 3.75	19.0 ± 4.30	0.780	0.772
	Post	18.0 ± 3.65	19.5 ± 4.27	**0.022**	**0.012**
Positive cognition	Pre	12.7 ± 2.88	13.3 ± 3.00	0.209	0.180
	Post	13.6 ± 3.33	13.2 ± 3.25	0.558	0.543
Interpersonal assistance	Pre	18.5 ± 3.74	19.0 ± 5.87	0.514	0.516
	Post	18.9 ± 3.04	18.9 ± 3.52	0.999	0.999
Family support	Pre	18.0 ± 3.15	17.4 ± 3.19	0.197	0.199
	Post	18.6 ± 3.01	18.0 ± 2.91	0.184	0.189

*P*-value1: tested by independent sample *t*-test; *P*-value2: Adjusted for gender, age, satisfaction with the relationship between parents, long-term roommates, family structure, how many good friends you have, dietary status, and physical activity. Values in bold indicate *p* < 0.05.

To further explore the distribution of well-being levels, we compared the proportions of students exceeding key score thresholds. At baseline, a significantly larger proportion of students in the control group scored above thresholds such as 12, 15, and 16 on positive emotion, and 13 or 14 on accomplishment (Supplementary Table S2). However, post-intervention, these differences disappeared. Importantly, the experimental group showed a significant increase in the proportion of students whose total well-being scores exceeded 60 after the intervention (Supplementary Table S3), suggesting that the intervention helped more students reach higher levels of psychological well-being, and this might be a potential threshold for future intervention effects assessment.

### 3.3 Resilience outcomes

Baseline scores on the resilience scale were similar between groups, with no significant differences in total or variable scores (Figure 1; Table 2). After the intervention, the emotional control score in the control group (19.2 ± 3.75) was significantly higher than that in the experimental group (18.0 ± 3.65).

Threshold analysis showed that at baseline, more students in the control group had goal focus scores above 21 and family support scores above 22 (Supplementary Table S4). After the intervention, the control group continued to show higher proportions of students with emotional control above 17 or 18. Meanwhile, the experimental group demonstrated significant improvements in the proportions of students scoring above 15, 18, and 21 on goal focus, above 17 on positive cognition, and above 91 on total resilience (Supplementary Table S5), indicating gains in specific areas of resilience.

### 3.4 Coping tendency outcome

Coping tendency scores between the control and experimental groups showed no significant changes before and after the intervention, as shown in Supplementary Table S8. The median coping tendency score in the control group slightly changed from 0.01 [−0.45, 0.33] pre-intervention to −0.01 [−0.29, 0.39] post-intervention (*p* = 0.905), and the experimental group’s score changed from −0.02 [−0.32, 0.33] to −0.13 [−0.36, 0.35] (*p* = 0.786). Neither the positive nor negative coping scores changed significantly in either group (all *p* > 0.05).

### 3.5 Paired population analysis

**Figure 2:**
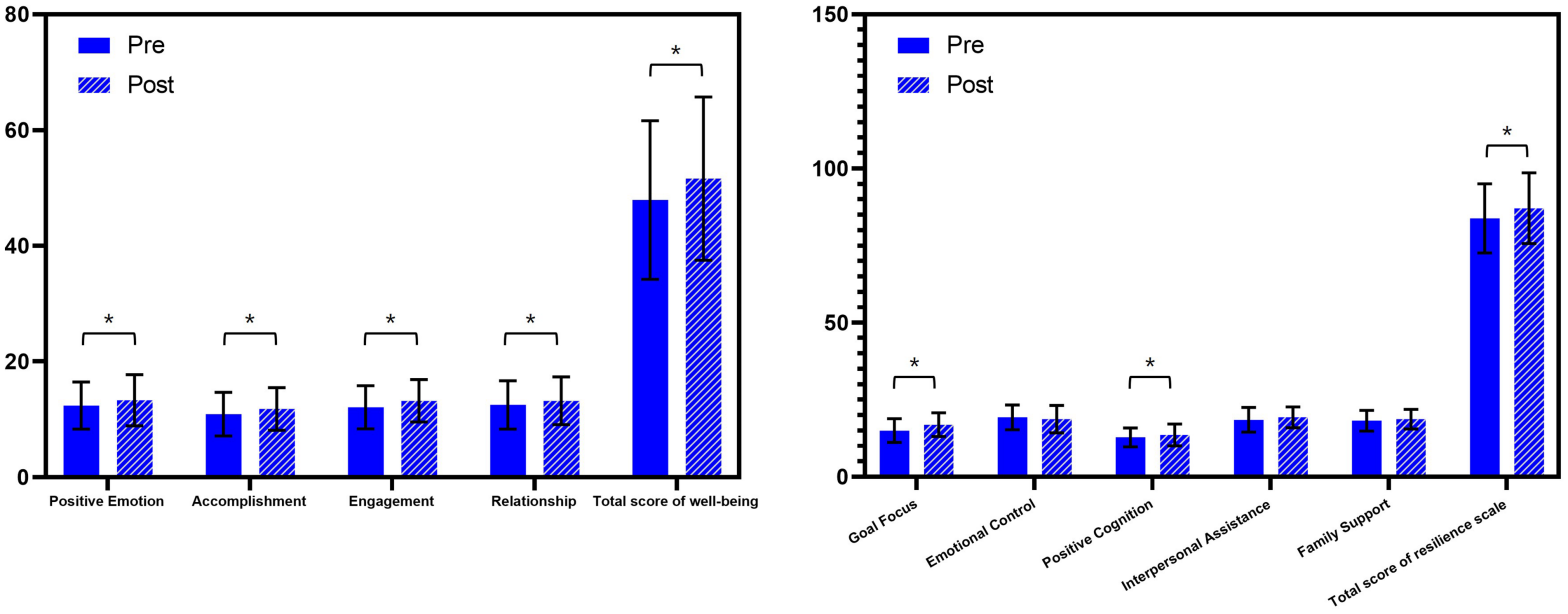
Scores of well-being (left) and resilience (right) of the experimental group pre and post intervention in the paired population (*N* = 58. Data are presented as mean and SD. *: significant difference before and after intervention tested by linear mixed effect model).

In the experimental group, 58 participants had both pre- and post-intervention test results that could be matched using random numbers (paired population). Significant improvements were observed in the scores for positive emotion, accomplishment, engagement, relationship, goal focus, positive cognition, total well-being, and total resilience in this paired population after adjusting for covariates (gender, age, satisfaction with parental relationships, cohabitation status, family structure, number of close friends, dietary habits, and physical activity levels) (Table 3; Figure 2).

**Table 3:** Pre–post comparison of scores within the paired experimental group (*N* = 58) using linear mixed-effect model.

Variable	Crude model		Adjusted model	
	β (SE)	*p*-value	β (SE)	*p*-value
Total score of well-being	3.69 (1.42)	**0.012**	5.93 (1.53)	**<0.001**
Positive emotion	0.90 (0.48)	0.067	1.63 (0.51)	**0.002**
Accomplishment	0.93 (0.44)	**0.038**	1.32 (0.50)	**0.011**
Engagement	1.16 (0.47)	**0.016**	1.52 (0.51)	**0.004**
Relationship	0.71 (0.45)	0.124	1.52 (0.45)	**0.001**
Total score of resilience	3.33 (1.30)	**0.013**	3.81 (1.39)	**0.008**
Goal focus	1.83 (0.48)	**<0.001**	2.14 (0.52)	**<0.001**
Emotional control	−0.55 (0.44)	0.217	−0.52 (0.49)	0.290
Positive cognition	0.81 (0.46)	0.080	1.08 (0.48)	**0.030**
Interpersonal assistance	0.79 (0.51)	0.127	0.74 (0.55)	0.177
Family support	0.45 (0.37)	0.235	0.44 (0.41)	0.292

Adjusted model corrects for gender, age, satisfaction with the relationship between parents, long-term roommates, family structure, how many good friends you have, dietary status, and physical activity. Values in bold indicate *p* < 0.05.

However, no significant changes were observed in positive or negative coping tendencies in the paired population following the intervention (Supplementary Table S6).

## 4 Discussion

Middle school students are in a critical stage of adolescence, characterized by increasing academic pressure and evolving self-cognition. The rising prevalence of adolescent depression, often with early-onset and recurrent patterns, is linked to long-lasting psychosocial impairments that can persist into adulthood (Wilson and Dumornay, 2022). Despite the pressing importance of addressing these issues, mental health interventions grounded in PERMA theory remain scarce among Chinese middle school students. This study implemented a personalized mental health intervention informed by a prior needs assessment of junior high school students and found significant improvements in total well-being scores and four dimensions of well-being: positive emotion, accomplishment, engagement, and relationship. Furthermore, goal focus, positive cognition, and the total resilience score also showed significant increases post-intervention. At baseline, the control group scored higher on positive emotion, relationship, and total well-being compared to the experimental group, but these differences were no longer significant after the intervention, demonstrating the intervention’s effectiveness in bridging the gap between the groups.

The intervention effectively improved well-being across all measured components. Positive emotions, such as pride, joy, and contentment, are particularly relevant in collective societies where individuals strive to align with the expectations of family, friends, and peers (Lambert D’raven and Pasha-Zaidi, 2015). Experiencing positive emotions can enhance students’ motivation for daily studies, improve relationships with classmates and family, and contribute to an overall positive class atmosphere (Green, 2022). Engagement, which encourages independent thinking and resilience, fostered a sense of accomplishment in students. The intervention helped students set realistic goals and cope with setbacks, promoting values of belonging and interdependence (Green et al., 2019; Uchida and Ogihara, 2012).

At baseline, the experimental group had significantly lower relationship scores, suggesting challenges in parental and peer relationships, which were highlighted in the pre-course needs assessment. The intervention program addressed these issues by teaching students to think from others’ perspectives, establish and protect psychological boundaries, express their feelings, opinions, and requests positively, respond actively to others’ communication needs, and genuinely care for others to foster peer friendships. These strategies effectively improved the relationship scores in the experimental group after adjusting for potential covariates, rendering their scores comparable to those of the control group after the intervention. Accomplishment often hinges on academic success and proficiency in skills that can earn admiration from peers for junior high school students. The intervention course not only helped students identify their strengths but also guided them in setting realistic goals for different life stages. By achieving these goals, students experienced an enhanced sense of accomplishment, which motivated them to strive for personal growth and success (Metrat-Depardon and Teo, 2021).

Resilience, a key factor in adapting to adversity, was significantly enhanced in terms of goal focus, positive cognition, and total resilience after intervention. Students in the experimental group became more focused persistent under high pressure, likely due to the intervention’s emphasis on goal-setting and resilience-building. However, the emotional control scores in the experimental group post-intervention were statistically lower than those in the control group. This may be attributed to the timing of the intervention, which concluded close to the final exam period. The increased academic stress during this time might have hindered students’ ability to regulate emotions effectively, highlighting the complex relationship between cognitive focus and emotional control (Turnbull and Salas, 2021). This finding underscores the importance of designing multi-dimensional interventions that address both cognitive and emotional aspects of resilience.

Regarding coping styles, no significant changes were observed in any of the indicators after the intervention, although the experimental group showed a slight increase in positive tendency and a slight decrease in negative tendency. This may be due to the need to standardize coping style scores, which could have limited the magnitude of observable changes. Another reason might be the relatively short duration of the intervention, as coping mechanisms often require longer-term strategies or different approaches to show measurable changes. Defining coping styles for different individuals is inherently challenging, as some coping strategies incorporate predominantly positive elements, such as seeking support and actively attempting to change the situation, while others involve predominantly negative elements, such as avoidance and venting (Dijkstra and Homan, 2016). It is important to note that the labels “positive” and “negative” are relative. Positive coping methods do not always lead to favorable outcomes, nor do negative coping methods necessarily result in unfavorable consequences. For instance, strategies like “accepting reality” and “self-comfort” are classified as negative coping methods, yet they can effectively mitigate the emotional impact of frustration (Xie, 1998). This highlights the need for further in-depth and longer period research to better understand the nuanced effects of coping styles across diverse situations and populations.

The targeted PPI, designed based on pre-course surveys, demonstrated significant intervention effects, aligning with some previous studies. For example, a Singapore study based on the PERMA model showed significant well-being improvements in students post-intervention, though not significantly exceeding the control group (Metrat-Depardon and Teo, 2021). In contrast, an American study involving 9th and 12th grade students found that an 8-week PPI had no significant effects on anxiety, depression, spiritual well-being, or happiness (Eisenreich, 2023), a disparity potentially attributed to the latter’s lack of needs assessment and tailored design. This study highlights how targeted PPIs—informed by pre-assessment—can better support adolescents (Metrat-Depardon and Teo, 2021). Moreover, effective positive education programs can indirectly improve teacher enthusiasm and student-teacher relationships (Zheng, 2021). Nevertheless, success depends critically on stakeholder involvement: implementation requires active participation from all school staff and parental support. Overburdened personnel or unsupportive parents can significantly hinder effectiveness. In addition, the positive results of this study remained significant after adjusting for potential covariates, including sociodemographic characteristics, diet, and physical activity, etc., ensuring that the effects observed in the study were not confounded.

The effectiveness of the intervention might have been influenced by cultural values prioritizing academic success and collective well-being. In Chinese culture, relationships and achievement are often seen as interconnected, and students may place more emphasis on academic and social harmony than on individual success, which could amplify or constrain the intervention’s effects (Lambert D’raven et al., 2015). This cultural context likely shaped students’ responses to the intervention, as they may have been more motivated by the need to meet family and peer expectations than by personal psychological growth. While this study offers valuable insights into the effectiveness of a personalized PERMA-based intervention, its findings may not be fully generalizable beyond Shanghai. Socioeconomic diversity, rural–urban differences, and variations in educational systems could influence the outcomes. For instance, students in rural areas may face different stressors and support structures compared to their urban counterparts, which could affect the intervention’s impact. Further research is needed to explore these factors and test the intervention’s scalability in diverse contexts.

Although this study is among the few in China to demonstrate the effectiveness of a personalized PERMA-based intervention for junior high school students, it has several limitations. First, due to the study design or students’ potential concerns about disclosing personal information, only 58 participants in the intervention group could be matched with pre- and post-intervention questionnaire results using random numbers. Along with the limited total participants, this reduced the statistical power of the analysis. Second, the intervention concluded during the school’s final exam period, which likely influenced students’ mental states and their responses to the questionnaires. Finally, while the results of this pilot study are promising, the short intervention and follow-up period leaded to the need for caution in interpreting the results. Longer follow-up studies are warranted to confirm the sustained effects of personalized PPIs on students’ long-term mental health.

## 5 Conclusion

This personalized mental health intervention study, grounded in the PERMA theory, demonstrated significant improvements in positive emotion, accomplishment, engagement, relationship, overall well-being, goal focus, positive cognition, and resilience following the implementation of the PPI. By guiding and coaching students in integrating PPI principles into their daily lives, the intervention effectively enhanced their motivation for learning and improved their interpersonal relationships. Future studies with larger sample sizes and extended follow-up periods are needed to fully explore and confirm the long-term benefits of this intervention.

## Data Availability

The raw data supporting the conclusions of this article will be made available by the authors, without undue reservation.
