# Supplementary material for: Effects of a personalized PERMA-based intervention on the mental health of junior high school students: a randomized clinical trial
Source: Front Psychol. 2025 Aug 29;16:1535744. doi: 10.3389/fpsyg.2025.1535744 (PMC12426034; doi:10.3389/fpsyg.2025.1535744)
Supplement: Supplementary file 1 [file Supplementary_file_1.DOCX]

**Supplementary Material**

**Table S1 Standard program schedule**

| Activity No. | Subject | Activity goal | Activity contents | Methods | Time period |
| --- | --- | --- | --- | --- | --- |
| 1 | Introduction +Positive Emotions | 1. Understand the purpose, content, methods and requirements of this intervention activity  2. Through this activity, students can have a basic understanding of emotions and improve their insight into emotions  3. Understand their own emotional characteristics and understand the impact of emotions on personal behavior and life | 1. Introduce the activity plan (goals, content, methods, duration)  2. Activity requirements (confidentiality, expectations, homework, self-evaluation)  3. Personalized needs survey  4. Warm-up activity: Strong wind  5. Theme activity: Emotional kaleidoscope  (1) Emotional face  (2) You act, I guess  (3) Understanding emotions  6. Review and summary  7. Homework: Record "My Emotional Barometer" every day for a week | 1. Lecture method  2. Lecture method  3. Questionnaire method  4. Game interaction  5. Questioning method. Group discussion method  6. PPT lecture  7. Lecture method | 1. 2min  2. 3min  3. 5min  4. 5min  5. 20min  6. 5min  7. 5min |
| 2 | Positive Emotions | 1. Through this activity, students can pay attention to other people's feelings and learn to express emotions appropriately and timely  2. Be able to deal with negative emotions and improve the ability to deal with negative emotions  3. Be familiar with ways to maintain positive emotions and vent negative emotions. | 1. Homework review  2. Warm-up activity: Grab the stool  3. Theme activity: Be the master of your emotions  (1) Emotion test  (2) Let it all out  (3) I am the master of my emotions  (4) Case analysis 1  4. Review and summary  5. Homework: Record three good things every day for a week | 1. Discussion method  2. Game interaction  3. Questioning method. Group discussion method  4. PPT teaching  5. Lecture method | 1. 5min  2. 5min  3. 25min  4. 5min  5. 5min |
| 3 | Positive Engagement | 1. Under the guidance of the instructor, you can use mindfulness to experience the present moment  2. Under the guidance of the instructor, learn to use concentration | 1. Homework review  2. Warm-up activity: Fun counting  3. Theme activity: The secret of concentration  (1) Characteristics of concentration  (2) Concentration diagnosis book  (3) Case analysis 2  4. Review and summary  5. Homework: Do half an hour of concentration exercises every day for a week | 1. Discussion method  2. Game interaction  3. Lecture method  . Questioning method. Group discussion method  4. PPT lecture  5. Lecture method | 1. 5min  2. 5min  3. 25min  4. 5min  5. 5min |
| 4 | Positive Engagement | 1. Under the guidance of the instructor, be able to use games to experience flow  2. Understand how to generate happiness during the learning process  3. Be familiar with the "Five-step Escape Method" | 1. Homework review  2. Warm-up activity: Catching dragonflies  3. Theme activity: Concentration improvement plan  (1) Concentration training: Schulte grid  (2) Make suggestions  (3) Case analysis 3  4. Review and summary  5. Homework: Five-step escape method | 1. Discussion method  2. Game interaction  3. Game interaction. Brainstorming. Group discussion method. Case analysis  4. PPT teaching  5. Lecture method | 1. 5min  2. 5min  3. 20min  4. 5min  5. 10min |
| 5 | Positive Relationships | 1. Understand effective expression  2. Be familiar with how to get along with parents  3. Master effective expression methods | 1. Homework review  2. Warm-up activity: Paper tearing instructions  3. Theme activity: How to get along with parents  (1) Clean up the "garbage" of mood  (2) Reveal the secrets of parent-child relationship  (3) Effective communication training  (4) The battle between father (mother) and son (daughter) (Case analysis 4)  4. Review and summary  5. Homework:  (1) Share the "Three Steps of Effective Expression" with parents  (2) Write a "Thank You Letter" within a week | 1. Discussion method  2. Game interaction  3. Game interaction. Group discussion method. Lecture method. Scenario practice method  4. PPT lecture  5. Lecture method | 1. 5min  2. 5min  3. 25min  4. 5min  5. 5min |
| 6 | Positive Relationships | 1. Understand your own friendship characteristics  2. Be able to self-assess your friendship skills  3. Under the guidance of a mentor, be familiar with methods to maintain, develop, and improve peer relationships | 1. Homework review  2. Warm-up activity: Say something flattering  3. Theme activity: My flower of friendship  (1) Draw my flower of friendship  (2) Share. Show my flower of friendship  (3) Case analysis 5  4. Review and summary  5. Homework: Improve interpersonal relationship worksheet | 1. Discussion method  2. Game interaction  3. Game interaction. Group discussion method. Case analysis  4. PPT teaching  5. Lecture method | 1. 5min  2. 5min  3. 25min  4. 5min  5. 5min |
| 7 | Positive Meaning | 1. Through this activity, learn more meaningful things and discover the positive meaning of life  2. By reviewing the major life events in memory, find the reasons for personal optimism or pessimism, change unreasonable cognitive beliefs, and achieve the purpose of improving self-evaluation | Homework review  2. Warm-up activity: Five steps to growth  3. Theme activity: My rainbow of life  (1) My lifeline  (2) Case analysis 6  4. Review and summary  5. Homework: Do something that you think is meaningful | 1. Discussion method  2. Game interaction 3. Game interaction. Group discussion method. Case analysis  4. PPT teaching  5. Lecture method | 1. 5min  2. 10min  3. 20min  4. 5min  5. 5min |
| 8 | Positive Achievements + concluding | 1. Through this activity, students can understand more about their own strengths  2. They can correctly set life goals at different stages  3. Understand what students have gained from the activity  4. Students can understand the benefits of positive psychology and apply the methods and techniques of positive psychology in their future lives | 1. Homework review  2. Warm-up activity: Advantage bombing  3. Theme activity: Life pyramid  (1) 20 selves  (2) Case analysis 7  4. Students share their course gains  5. Tutor's course summary. Acknowledgements | 1. Discussion method  2. Game interaction  3. Practice method. Brainstorming method. Group discussion. Case analysis  4. Discussion method  5. PPT teaching | 1. 5min  2. 10min  3. 20min  4. 10min  5. 5min |

**Table S2 P value for comparison of the proportion of well-being scores exceeding the threshold between the experimental group and the control group**

| **Threshold** | **Pre-Intervention** | | | |  | **Post-Intervention** | | | |
| --- | --- | --- | --- | --- | --- | --- | --- | --- | --- |
|  | **Positive Emotion** | **Accomplishment** | **Engagement** | **Relationship** |  | **Positive Emotion** | **Accomplishment** | **Engagement** | **Relationship** |
| 10 | 0.843 | 0.999 | 0.858 | 0.228 |  | 0.331 | 0.719 | 0.191 | 0.999 |
| 11 | 0.266 | 0.999 | 0.999 | 0.076 |  | 0.999 | 0.868 | 0.058 | 0.999 |
| 12 | **0.042** | 0.257 | 0.516 | 0.129 |  | 0.863 | 0.999 | 0.742 | 0.999 |
| 13 | 0.193 | **0.042** | 0.870 | 0.420 |  | 0.257 | 0.615 | 0.999 | 0.745 |
| 14 | 0.052 | **0.047** | 0.394 | 0.333 |  | 0.747 | 0.860 | 0.999 | 0.517 |
| 15 | **0.021** | 0.331 | 0.212 | 0.072 |  | 0.510 | 0.575 | 0.612 | 0.739 |
| 16 | **0.015** | 0.662 | 0.999 | 0.304 |  | 0.734 | 0.296 | 0.191 | 0.865 |
| 17 | 0.059 | 0.764 | 0.818 | 0.331 |  | 0.999 | 0.587 | 0.830 | 0.999 |
| 18 | **0.044** | 0.717 | 0.367 | 0.204 |  | 0.999 | 0.531 | 0.999 | 0.839 |
| 19 | 0.210 | 0.999 | 0.212 | 0.123 |  | 0.999 | 0.999 | 0.779 | 0.802 |
| **Total score of well-being** | | | | | | | | | |
| 60 | 0.064 | | | |  | 0.390 | | | |
| 65 | 0.144 | | | |  | 0.999 | | | |
| 70 | 0.999 | | | |  | 0.999 | | | |
| 75 | 0.999 | | | |  | 0.999 | | | |

Note: comparison using chi-square test.

**Table S3 P value for comparison of the proportion of well-being scores exceeding the threshold before and after the intervention in the experimental group**

| **Threshold** | **Positive Emotion** | **Accomplishment** | **Engagement** | **Relationship** |
| --- | --- | --- | --- | --- |
| 10 | 0.688 | 0.223 | 0.341 | 0.560 |
| 11 | 0.858 | 0.252 | 0.117 | 0.390 |
| 12 | 0.320 | 0.257 | 0.869 | 0.317 |
| 13 | 0.999 | 0.379 | 0.625 | 0.519 |
| 14 | 0.328 | 0.138 | 0.237 | 0.999 |
| 15 | 0.493 | 0.249 | 0.080 | 0.999 |
| 16 | 0.205 | 0.296 | 0.131 | 0.726 |
| 17 | 0.174 | 0.400 | 0.505 | 0.249 |
| 18 | 0.144 | 0.327 | 0.103 | 0.505 |
| 19 | 0.142 | 0.439 | 0.122 | 0.562 |
|  | **Total score of well-being** | | | |
| 60 | **0.019** | | | |
| 65 | 0.382 | | | |
| 70 | 0.400 | | | |
| 75 | 0.363 | | | |

Note: comparison using chi-square test.

**Table S4 P value for comparison of the proportion of resilience scale exceeding the threshold between the experimental group and the control group**

| **Threshold** | **Pre-Intervention** | | | | |  | **Post-Intervention** | | | | |
| --- | --- | --- | --- | --- | --- | --- | --- | --- | --- | --- | --- |
|  | **Goal Focus** | **Emotional Control** | **Positive Cognition** | **Interpersonal Assistance** | **Family Support** |  | **Goal Focus** | **Emotional Control** | **Positive Cognition** | **Interpersonal Assistance** | **Family Support** |
| 15 | 0.744 | 0.811 | 0.587 | 0.999 | 0.607 |  | 0.472 | 0.519 | 0.402 | 0.999 | 0.211 |
| 16 | 0.519 | 0.410 | 0.672 | 0.341 | 0.249 |  | 0.254 | 0.321 | 0.711 | 0.472 | 0.210 |
| 17 | 0.999 | 0.581 | 0.161 | 0.602 | 0.863 |  | 0.105 | **0.012** | 0.835 | 0.694 | 0.999 |
| 18 | 0.145 | 0.299 | 0.327 | 0.622 | 0.999 |  | 0.734 | **0.038** | 0.802 | 0.723 | 0.999 |
| 19 | 0.421 | 0.871 | 0.999 | 0.872 | 0.739 |  | 0.350 | 0.076 | 0.999 | 0.519 | 0.406 |
| 20 | 0.282 | 0.747 | 0.999 | 0.999 | 0.852 |  | 0.839 | 0.247 | 0.999 | 0.328 | 0.719 |
| 21 | **0.030** | 0.620 |  | 0.729 | 0.382 |  | 0.999 | 0.056 |  | 0.860 | 0.282 |
| 22 | 0.133 | 0.999 |  | 0.701 | **0.040** |  | 0.999 | 0.124 |  | 0.568 | 0.231 |
| 23 | 0.677 | 0.672 |  | 0.400 | 0.999 |  | 0.999 | 0.296 |  | 0.999 | 0.060 |
| 24 | 0.677 | 0.366 |  | 0.607 | 0.677 |  | 0.999 | 0.327 |  | 0.999 | 0.677 |
| 25 | 0.999 | 0.744 |  | 0.327 | 0.999 |  | 0.612 | 0.246 |  | 0.999 | 0.999 |
| 26 |  |  |  | 0.677 |  |  |  |  |  | 0.477 |  |
| 27 |  |  |  | 0.999 |  |  |  |  |  | 0.999 |  |
| 28 |  |  |  | 0.999 |  |  |  |  |  | 0.999 |  |
| 29 |  |  |  | 0.999 |  |  |  |  |  | 0.999 |  |
| **Total score of resilience scale** | | | | | | | | | | | |
| 80 | 0.860 | | | | |  | 0.216 | | | | |
| 85 | 0.052 | | | | |  | 0.747 | | | | |
| 90 | 0.191 | | | | |  | 0.366 | | | | |
| 95 | 0.304 | | | | |  | 0.999 | | | | |
| 100 | 0.367 | | | | |  | 0.999 | | | | |
| 105 | 0.999 | | | | |  | 0.999 | | | | |
| 110 | 0.999 | | | | |  | 0.612 | | | | |

Note: comparison using chi-square test.

**Table S5 P value for comparison of the proportion of resilience scale exceeding the threshold before and after the intervention in the experimental group**

| **Threshold** | **Goal Focus** | **Emotional Control** | **Positive Cognition** | **Interpersonal Assistance** | **Family Support** |
| --- | --- | --- | --- | --- | --- |
| 15 | **0.018** | 0.267 | 0.058 | 0.304 | 0.327 |
| 16 | 0.075 | 0.228 | 0.116 | 0.116 | 0.093 |
| 17 | 0.105 | 0.080 | **0.014** | 0.212 | 0.366 |
| 18 | **0.049** | 0.125 | 0.082 | 0.182 | 0.192 |
| 19 | 0.124 | 0.053 | 0.999 | 0.747 | 0.869 |
| 20 | 0.282 | 0.101 | 0.999 | 0.416 | 0.466 |
| 21 | **0.030** | 0.113 |  | 0.602 | 0.999 |
| 22 | 0.327 | 0.087 |  | 0.999 | 0.999 |
| 23 | 0.677 | 0.999 |  | 0.999 | 0.400 |
| 24 | 0.999 | 0.792 |  | 0.999 | 0.999 |
| 25 | 0.999 | 0.999 |  | 0.999 | 0.999 |
| 26 |  |  |  | 0.477 |  |
| 27 |  |  |  | 0.999 |  |
| 28 |  |  |  | 0.999 |  |
| 29 |  |  |  | 0.999 |  |
|  | **Total score of resilience scale** | | | | |
| 80 | 0.366 | | | | |
| 85 | 0.414 | | | | |
| 90 | 0.138 | | | | |
| 91 | **0.029** | | | | |
| 95 | 0.779 | | | | |
| 100 | 0.531 | | | | |
| 105 | 0.999 | | | | |
| 110 | 0.612 | | | | |

Note: comparison using chi-square test.

**Table S6 Simple Coping Style scores of control group and experimental group before and after intervention**

| **Scale** | **Control group** | | |  | **Experimental group** | | | **P-value 2** | **P-value 3** |
| --- | --- | --- | --- | --- | --- | --- | --- | --- | --- |
|  | **Pre-intervention** | **Post-intervention** | **P-value 1** |  | **Pre-intervention** | **Post-intervention** | **P-value 1** |  |  |
| **Whole population (N=154)** |  |  |  |  |  |  |  |  |  |
| Coping tendency | 0.01 [-0.45~0.33] | -0.01 [-0.29~0.39] | 0.905 |  | -0.02 [-0.32~0.33] | -0.13 [-0.36~0.35] | 0.786 | 0.951 | 0.680 |
| Positive tendency | 0.11 [-0.69~0.78] | 0.06 [-0.59~0.58] | 0.877 |  | -0.04 [-0.73~0.79] | 0.01 [-0.56~0.72] | 0.792 | 0.902 | 0.940 |
| Negative tendency | 0.15 [-0.62~0.58] | 0.02 [-0.61~0.54] | 0.770 |  | 0.07 [-0.71~0.74] | 0.04 [-0.54~0.51] | 0.857 | 0.877 | 0.857 |
| **Paired population (N=58)** |  |  |  |  |  |  |  |  |  |
| Coping tendency | - | - | - |  | -0.01 [-0.30~0.32] | -0.13 [-0.35~0.39] | 0.999 | - | - |
| Positive tendency | - | - | - |  | 0.01 [-0.67~0.74] | 0.04 [-0.53~0.68] | 0.999 | - | - |
| Negative tendency | - | - | - |  | 0.18 [-0.69~0.64] | 0.05 [-0.53~0.53] | 0.999 | - | - |

Note: Data are presented as Median [P25~P75]. P-value 1 was obtained for comparison of pre- and post-intervention scores using Wilcoxon test for whole population and paired t-test for paired population; P-value 2 was obtained for comparison of control group and experimental group scores before intervention using Wilcoxon test; P-value 3 was obtained for comparison of control group and experimental group scores after intervention using Wilcoxon test. -: not applicable.
